# The Practitioners’ Visiting List for 1911

**Published:** 1910-12

**Authors:** 


**The Practitioners' Visiting List** for 1911. An invaluable pocket-sized book containing memoranda and data important for every physician, and ruled blanks for recording every detail of practice. Price by mail, postpaid, to any address, $1.25. Thumb-letter index, 25 cents extra. Lea & Febiger, Publishers, Philadelphia and New York.

With its usual promptitude and in all the beauty of the printer's and binder's arts, this invaluable visiting list presents itself for use in 1911. It is made up in four styles, each having its special form of usefulness. The weekly, monthly, and 30-patient perpetual contain 32 pages of data and 160 pages of classified blanks. The 60-patient perpetual consists of 256 pages of blanks alone. Each is issued in one wallet-shaped book, bound in flexible leather, with flap and pocket, pencil with rubber, and calendar for two years. The preliminary text, containing many hints with reference to emergencies and to clinical needs, is of special importance. Among the subjects dealt with may be found thermometric scales; weights, measures, and the metric system; notes on the examination of urine; inmportant incompatibles; artificial respiration details; poisons and antidotes; table of doses; and therapeutic reminders. It is one of the better books of its kind. A descriptive circular showing the several styles and giving all necessary information will be sent by the publishers on request. J. A. R.

